# In-situ cell density monitoring and apoptosis detection in adherent Vero cell bioreactor cultures

**DOI:** 10.1186/1753-6561-5-S8-P8

**Published:** 2011-11-22

**Authors:** Emma Petiot, Amal El-Wajgali, Geoffrey Esteban, Cécile Gény, Hervé Pinton, Annie Marc

**Affiliations:** 1 Laboratoire Réactions et Génie des Procédés, UPR-CNRS 3349, Nancy-Université, Vandœuvre-lès-Nancy, France; 2 FOGALE nanotech, Nîmes, France; 3 Sanofi pasteur, Marcy L’Etoile, France

## Background

In cell-based processes, and particularly in viral vaccine production, cell growth and death are strategic informations to obtain for process monitoring (ie. scale-up, determination of MOI, TOI, and harvest time). Dielectric spectroscopy is a tool which was increasingly implemented on cell-culture bioreactors as it presents great potentials, compared to other methods, for the in-line monitoring of these two crucial parameters. Considering viral vaccine production, Vero cells are one of the most employed cell platform. But, due to its adherent characteristics, few in-line techniques were developed for cell density monitoring, on the contrary to the ones available for suspension cells. In addition, it should be underline that no in-line technique exists for quantification or detection of the mammalian cell death, despite the importance of this parameter for cell culture processes.

## Materials and methods

The Vero cell line employed in this study was provided by Sanofi Pasteur. Cells were cultivated in serum-free conditions; either in a reference medium or in a modified medium with alanine-glutamine peptide (Glutamax^®^) substituted to glutamine. The adhered cell population was numbered on haemacytometer after crystal violet treatment. The apoptotic cells, labelled with annexin V, were quantified by flow cytometry (Guava). The in-line recording of permittivities at different frequencies and of the characteristic frequency of the cell population, fc, were performed by a Fogale Biomass system^®^.

## Theoretical background

The application of the permittivity theory to mammalian cell density quantification was well described in previous studies [1]. For a basic comprehension it has to be precised that, in this method, viable cells are considered as miniature condensators, and permittivity measurement corresponds to the amplitude of the decharge curve of cell culture suspension. This curve modelling allowed to relate permittivity to different physical parameters, such as cell size, cell membrane capacitance or cell intracellular conductivity as described by the following equations [2]. From these mathematical relations, a parameter that will be called specific permittivity of the cells (Δε_fogale_ / C) was calculated. Most of these physical parameters could be potentially impacted by changes in the cell physiology and especially by modifications induced by cell death.

Δε = 9 . r . B . C_M_ / 4 = 3 . ð . r^4^ . C . C_M_

fc = σi / ( 2 . B . r . C_M_)

Δε: *permittivity*, *pF.cm^-1^*

*fc: characteristic frequency*, *Hz*

*B : biovolume* (*percentage of viable cell volume in the culture suspension*)

*C : cell concentration*, *cell.mL^-1^*

*r : cell radius*, *m*

*C_M_ : membrane capacitance*, *F. m^-2^*

*σi : intracellular conductivity*, *mS.cm^-1^*

## Results

### Dielectric properties of adherent Vero cells

**Figure 1:**
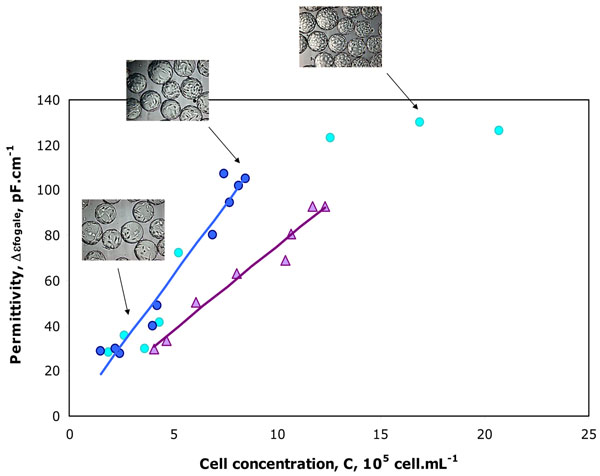
Evolution of the relative permittivity, Δε_fogale_, with the adhered Vero cell concentration and microscopic observations of microcarriers at different time points of the culture.

A valid correlation between biovolume and cell concentration was observed for batch cultures performed in both media, confirming that the Schwan model [3], originally developed for spherical cells, was also implementable to cells attached on microcarrier surface. The slope of the correlation between permittivity and cell concentration highlighted the impact of the medium composition on the Vero cell dielectric properties. Indeed, in the modified cell culture medium, the slope was lower suggesting a reduction of specific permittivity, and so potential changes in cell dielectric properties (C_M_ and σi) (Figure 1).

The Fogale Biomass system^®^ also allowed to observe the impact of cell density on cell morphology. Indeed, for culture in the reference medium, a linear correlation was observed under 10 x 10^5^ cell.mL^-1^. Beyond this cell density, the specific permittivity decreased indicating a decrease of the biovolume per cells. This was confirmed by microscopic observation at different time points of the culture, demonstrating a reduction of cell size due to carrier surface saturation. So, an accurate monitoring of Vero cells adhered on microcarriers could be realized with Fogale biomass system^®^, in different culture media and during the different exponential or decline culture phases.

### In-line detection of Vero cell death

The Fogale biomass system^®^ was also applied to in-line detect Vero cell death. We only focused on apoptosis, while no necrotic cells were detected in these Vero cell cultures. Dielectric spectroscopy parameters were plotted and correlated to apoptotic cell death occurrence. Thus, a drastic increase of cell apoptosis was observed to be concomitant with fc parameter increase, whatever the culture medium or the feeding process used (batch / fed-batch). Plotting the characteristic frequency derivative, dfc/dt, with the apoptotic cell concentration highlighted that this derivative always became equal to zero when the apoptosis occurred. As a proof-of-concept, an apoptotic inducer, the actinomycin D, was added during the exponential cell growth phase of a batch culture. In that case the fc parameter presented the same behaviour confirming the relationship between apoptosis physiological modifications and the physical parameters impacting fc (r, C_M_, σi).

## Conclusion

The first major impact of this work was to demonstrate that no model adaptation was needed to monitor adherent cell concentration by using permittivity measurements. An accurate monitoring of adhered Vero cell concentration was obtained with Fogale Biomass system^®^ until 1 x 10^6^ cell.mL^-1^. A further development of the method should be to monitor higher densities of adherent cells. This could be easily achieved by monitoring the cell size evolution and correcting the changes induced in the correlations. The second major impact of this work was to demonstrate the potentials of this method for the risk-mitigation strategies. Indeed, it was possible to detect a high increase of cell apoptosis in cell culture performed with reference operating conditions, but also, an abnormal increase of apoptotic cell concentration artificially induced during the culture process. This observation validates the fact that detection of apoptosis occurrence, due to viral infection for example, could be monitored with this system.
